# A Convert to the Voluntary System

**Published:** 1919-08-16

**Authors:** 


### MASONIC AID FOR HOSPITALS.

### A Convert to the Voluntary System.

The generosity of Freemasons was emphasised by the Chairman of the board of the Leicester Royal Infirmary at the laying of the foundation stone of its orthopædic department. On three separate occasions, he stated, the infirmary board received deputations from London asking it to erect such a department; on three occasions the plans were passed; and on three subsequent occasions the necessary permission to build was refused. The consequent temporary arrangements were inadequate, and now the Freemasons of Leicestershire and Rutland had come forward with a gift of £5,000 and desired to make the new department a memorial to those brethren who had fallen in the war. The gift was a lasting memorial to their generosity.

The foundation stone was laid with the ancient ceremonial of the Craft by the Provincial Grand Master, Mr. Edward Holmes, assisted by the Deputy Provincial Grand Master, Colonel C. F. Oliver, a member of the board of the infirmary, and other officers, including the House Governor and Secretary as Provincial Grand Registrar. The stone having been laid, the Provincial Grand Master placed grains of wheat upon the stone, saying, "We strew this corn as the emblem of plenty." He then sprinkled wine upon the stone, saying, "We pour this wine as the emblem of cheerfulness and joy." Next he poured oil upon the stone, saying, "We pour this oil as the emblem of prosperity and happiness," and lastly sprinkled salt with the words, "We sprinkle this salt as the emblem of wisdom, fidelity, and perpetuity." The Provincial Grand Master handed to the Chairman of the board a cheque for £5,000 with the earnest hope that in years to come many of their fellow-men would emerge from the doors of the department with limbs restored. The cheque was also evidence of their appreciation of the arduous and glorious work which their board, the doctors, nurses, and staff had discharged.

Mr. T. Fielding Johnson, jun., the Chairman, said that this generous gift showed better than all else the knowledge which the members of the Craft had of the requirements of medical science and their great interests in the welfare of others. The new department would be of great assistance to the institution, and undoubtedly the means of performing invaluable help not only to discharged soldiers.

The Mayor of the city compared the graciousness of the gift with the great work of the infirmary. He was sure that the infirmary would never lack practical means of support. "He used to be in favour of the municipalisation of hospitals and the nationalisation of other things, but his recent experiences had not strengthened him in that view." The memorial stone bears the following inscription in gilt letters:

"This stone was laid by Edward Holmes, Provincial Grand Master of Leicestershire and Rutland, on behalf of the Freemasons of the Province, who, in loving memory of those brethren who fell in the Great War, subscribed the sum of £5,000 towards the erection of this building, 28th July, 1919."

